# Nano-Engineered Sensor Systems for Disease Diagnostics: Advances in Smart Healthcare Applications

**DOI:** 10.3390/bios15120777

**Published:** 2025-11-26

**Authors:** Tianjun Ma, Jianhai Sun, Ning Xue, Jamal N. A. Hassan, Adeel Abbas

**Affiliations:** 1 School of Electronics and Communication Engineering, Quanzhou University of Information Engineering, Quanzhou 362000, China; matianjun@qzuie.edu.cn (T.M.); xuening@qzuie.edu.cn (N.X.); 2 State Key Laboratory of Transducer Technology Institute of Electronics, Chinese Academy of Sciences, Quanzhou 362000, China; sunjh@aircas.ac.cn; 3 General Education Centre, Quanzhou University of Information Engineering, Quanzhou 362000, China

**Keywords:** nanosensor, point-of-care diagnostics, biomedical monitoring, wearable sensor technology, molecular-level detection

## Abstract

Nano-engineered sensor systems represent a paradigm shift in disease diagnostics, offering unprecedented capabilities for precision medicine. This review methodically evaluates these advanced platforms, consolidating recent advancements across four critical clinical domains: diabetes monitoring, cancer detection, infectious disease diagnostics and cardiac/genetic health. We demonstrate how the unique properties of nanomaterials, such as graphene, quantum dots and plasmonic nanoparticles, are being harnessed to achieve remarkable gains in analytical sensitivity, selectivity and real-time monitoring. Specific breakthroughs include graphene-based sensors attaining clinically significant limits for continuous glucose monitoring, quantum dot bioconjugates enabling ultrasensitive imaging of cancer biomarkers and surface-enhanced Raman spectroscopy (SERS) probes facilitating early tumor identification. Furthermore, nanosensors exhibit exceptional precision in detecting viral antigens and genetic mutations, underscoring their robust translational potential. Collectively, these developments signal a clear trajectory toward integrated, intelligent healthcare ecosystems. However, for these promising technologies to transition into accessible and cost-effective diagnostic solutions, persistent challenges in scalability, manufacturing reproducibility and long-term biocompatibility must be addressed through continued interdisciplinary innovation.

## 1. Introduction

The living system is a complex supramolecular machine controlled by a variety of dynamic biological processes. Fluctuations in bioactivities and biomolecule concentrations convey vital information that aids in the understanding of primary molecular mechanisms and has substantial implications for medical applications [1,2]. Recently, sensors capable of detecting and monitoring functions in biological systems become essential research tools, addressing fundamental questions about the self-regulation of living organisms [3,4,5,6]. Additionally, these sensors have found commercial applications as key components in wearable devices that track vital signs in real time [7]. Sensors consist of two significant elements: a receptor moiety that identifies the biological target or its activity and a signal transduction element that translates this recognition into a measurable readout [8]. Sensors designed for in vitro and in vivo uses should ideally meet several criteria, including a dynamic sensing range that corresponds to the physiological levels of the analyte of interest, stability during the measurement period, rapid response times, high biocompatibility, high selectivity against main interferents in the biological environment, and minimal disruption to regular biological roles. Nanosensors, in particular, have lately emerged as intriguing candidates for meeting many of these strict criteria [9,10].

**Figure 1:**
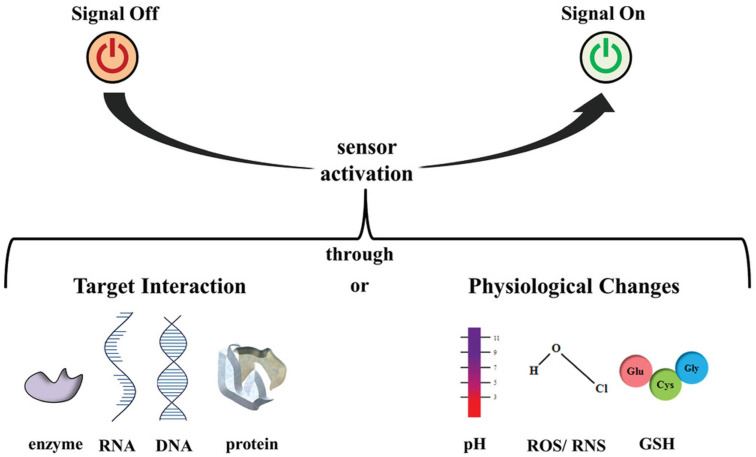
Overview of various approaches to activate nanosensor through enzymes, RNA, DNA, proteins, pH, ROS/RNS, or GSH interactions (Reprinted with permission from Ref. [12]. Copyright 2020 John Wiley & Sons).

Driven by advancements in nanotechnology, nanosensors have garnered attention for their notable sensing accuracy across several applications. Their small size, high sensitivity, and precision make them excellent for healthcare applications that need real-time health monitoring, early illness identification, and targeted drug delivery [11]. Chemical nanosensors find use in detecting diverse substances for pollution control, pharmaceutical development, environmental testing, identifying organophosphorus compounds, and diagnostic tools. Nanosensors can be categorized in multiple ways. One common approach is to classify them as biological, physical, or chemical. Biological nanosensors often pair a bio-receptor with a transducer. Fluorescence is a favored transduction technique due to its sensitivity and ease of use. Measurement strategies include binding active nanoparticles to intracellular proteins, engineering indicator proteins via site-directed mutagenesis for real-time analysis, or designing nanomaterials with specific bio-receptor attachment points. While electrochemical nanosensors can assess intracellular features, they typically lack the target specificity offered by bio-receptors (e.g., antibodies, DNA, or enzymes) found in biological sensors. Physical nanosensors measure properties like force and temperature, converting them into measurable signals. Activatable nanosensors represent a promising technology platform for the efficient detection and grading of diseases, even at early stages (Figure 1).

This review emphasizes the evolving design strategies of nanosensors by examining their activation mechanisms and clinical relevance. It begins with sensors triggered by specific target interactions, either through biocatalytic reactions or molecular binding events, followed by those responsive to physiological changes such as pH, oxidative stress, or temperature fluctuations. This work included peer-reviewed primary research articles that provided experimental validation and quantitative performance data (e.g., limit of detection, sensitivity, specificity) for nanosensors in biomedical applications. For each eligible study, we systematically extracted data on the sensor design, nanomaterial composition, underlying detection mechanism, and key analytical figures of merit. These activation pathways are directly linked to diagnostic performance, influencing sensitivity, specificity, and real-time responsiveness in complex biological environments. To illustrate their utility in healthcare, the review integrates detection methodologies including optical imaging, magnetic resonance imaging, magnetic particle spectroscopy, enzyme-linked immunosorbent assays, and lateral flow formats, each contextualized within the framework of nanosensor activation and its role in disease monitoring and point-of-care diagnostics [13].

## 2. Nanosensor Concepts and Mechanism of Action

### 2.1. Core Concepts of Nanosensor Technology

Nanosensors are miniature devices that detect physical, chemical, or biological changes at the nanoscale. They are typically classified as nano-physical, nano-chemical, or nano-biological sensors, depending on the nature of the analyte and the transduction mechanism. These sensors leverage high surface-to-volume ratios to achieve exceptional sensitivity and selectivity, making them ideal for real-time biomedical monitoring [14].

**Figure 2:**
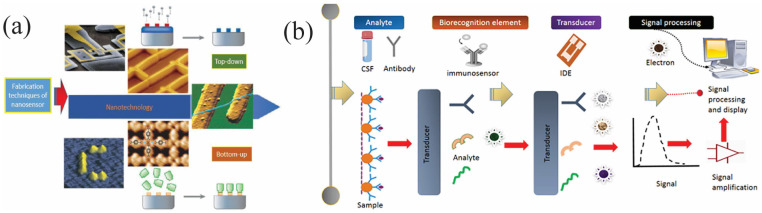
(**a**) Comparative overview of top-down and bottom-up nanostructure synthesis methods (Reprinted with permission from Ref. [16]. Copyright 2005 Springer Nature) and (**b**) the essential principles underlying nanosensors (Reprinted with permission from Ref. [13]. Copyright 2024 Elsevier).

Two primary approaches to nanostructure synthesis have evolved. These manufacturing strategies, known as top-down and bottom-up, differ significantly in terms of quality, speed, and cost. Figure 2a provides a general overview of the top-down and bottom-up concepts, along with various methods used to synthesize nanoparticles using these techniques. This provides a perfect idea for further exploring the process of “nanosensors”, “nanorobots”, or “micro- nano assemblies” in medical applications [15].

Nanosensors function by detecting changes in physical or chemical properties at the nanoscale and convert these changes into measurable signals. These devices, often constructed from nanoparticles, graphene, or nanowires, leverage the unique properties arising from their small size and large surface area. This interaction generates a physicochemical change that is then translated into a quantifiable effect, such as an optical or electrical signal.

Nanosensor design prioritizes sensitivity and selectivity towards the target analyte, enabling the detection and measurement of events occurring at the nanoscale. Miniaturization is another crucial principle in nanosensor development. Various nanosensor forms, including thin films, nanoscale wires, metal and metal oxide nanoparticles, carbon nanotubes, and polymer nanosensors, offer distinct properties and advantages for diverse sensing applications [17].

Beyond the manipulation and application of nanosensors, signal transduction represents another fundamental principle. Nanosensors typically comprise an analyte interaction element, a transducer, a signal processing unit, and a display system. Technological progress has yielded nanosensors capable of revolutionizing medical technology by providing real-time, quantitative dimensions of the human biochemical signaling network, paving the way for new diagnostic and therapeutic approaches [18]. In essence, nanosensors are vital for precise detection and measurement of properties or analytes at the nanoscale, revealing high accuracy and selectivity, capable of detecting even minute changes or concentrations of the target analyte (Figure 2b).

### 2.2. Nanosensor Design and Engineering

#### 2.2.1. Nanomaterial Properties Relevant to Sensing

Nanomaterials used in sensor platforms exhibit unique physicochemical properties that enhance sensitivity, selectivity, and signal transduction. Quantum dots offer tunable fluorescence, while graphene and carbon nanotubes provide high electron mobility and mechanical strength. Magnetic nanoparticles enable targeted analyte capture and signal amplification. These properties are critical for optimizing nanosensor performance across biomedical applications.

Advances in nanomaterial manipulation, fabrication, and characterization have profoundly influenced the area of nanosensors. These small devices, engineered and built at the nanoscale, are designed to discover and measure specific physical. Nanosensors exploit the unique properties of nanoscale materials to achieve high selectivity, sensitivity, and portability in sensing uses. A key aspect of nanosensor fabrication and design is the incorporation of materials like quantum dots, magnetic nanoparticles, graphene, and carbon nanotubes, selected for their excellent attributes such as high surface area, efficient electron transport and characteristic fluorescence. Metal nanoparticles, particularly gold and silver, are widely used in nanosensor platforms due to their strong surface-enhanced Raman scattering (SERS) properties, enabling highly sensitive detection of biological and chemical analytes. Carbon-based nanomaterials, including graphene, carbon nanotubes, and fullerenes, offer exceptional electrical, mechanical, and thermal characteristics, making them ideal for signal transduction and sensor miniaturization. These materials are frequently integrated into biosensor designs to enhance sensitivity, stability, and multiplexing capabilities. Researchers are integrating these nanomaterials into sensors to enhance surface area, increase catalytic activity, and increase overall sensing abilities [19].

#### 2.2.2. Fabrication and Engineering Strategies for Nanosensors

The fabrication of nanosensors involves precise engineering techniques to integrate functional nanomaterials into device architectures. Approaches such as template-assisted growth, directed self-assembly, and nanolithography enable controlled patterning and reproducibility. Top-down methods offer structural precision, while bottom-up strategies facilitate molecular-level assembly. Table 1 summarizes key fabrication techniques and their associated outcomes. Nanosensors are often fabricated and designed using optical signal transduction systems, empowering the detection and measurement of target analytes through optical signals such as absorbance, fluorescence or scattering. To improve the performance and functionality of nanosensors, researchers must first identify the specific target analyte.

Subsequently, appropriate transducer elements, sensing elements, and signal detection methods are selected based on the particular requirements of the application [20]. Nanosensors with high surface-to-volume ratios, such as metallic nanoparticles or quantum dots, generally are selected when high sensitivity and selectivity are paramount [21]. Furthermore, the shape and size of the nanosensor must be carefully optimized to ensure effective analyze detection and capture. The fabrication process involves assembling and synthesizing nanosensor components using techniques such as self-assembly, chemical deposition, and electrochemical methods [22]. As illustrated in Figure 2a, both top-down and bottom-up methodologies are used in nanotechnology, with top-down approaches generating structures through masked or maskless techniques, and bottom-up approaches incorporating self-organization of species and solid-state architectures, distinguishing between pure self-assembly and preset patterns [23].

Nanofabrication is therefore critical to the development and successful application of nanosensors. While conventional methods like e-beam, focused ion beam (FIB), and photolithography exist, they often suffer from limitations in cost, time consumption and scalability due to their imaging nature and serial writing. Nanoimprint lithography (NIL) presents a promising alternative, enabling the generation of nanoscale patterns at a lower cost, with higher throughput. The creation of nanoscale structures relies on two primary approaches in modern materials fabrication: top-down and bottom-up. Top-down methods utilize conventional techniques, such as electron beam lithography or optical, to remove additional material, leaving only the desired nanostructure. Conversely, bottom-up practices employ small atomic or molecular building blocks that self-assemble into the final structure through covalent bonds or van der Waals forces.

Nanotechnology uses often leverage both top-down and bottom-up techniques, depending on factors such as material possessions, compatibility, scale, accuracy, consistency, and device operational conditions. Regardless of ongoing challenges related to reproducibility and recognition limits, the continuing development of nanosensors holds significant promise for a wide range of applications.

### 2.3. Attributes of Nanosensor Technology

Nanosensors are devices that utilize the unique properties of nanoparticles and nanomaterials to detect and measure nanoscale events. The small size and large surface area of nanosensors result in improved selectivity and sensitivity, making them invaluable for a wide range of applications (Table 2). Nanosensors provide rapid, accurate and cost-effective data by identifying and quantifying target analytes in numerous samples, rendering them particularly well suited for precision agriculture. They also offer multiplexing proficiency, enabling the simultaneous detection of multiple target analytes. The ability of nanosensors to interact directly with atoms and molecules results in ultra-high sensitivity in detection. These properties have facilitated significant advances in metabolite detection and monitoring, making them ideally suited for fast, point-of-need uses.

Nanosensors are also well suited for high-throughput and multiplexed sensing uses due to their potential for massive scale incorporation into addressable arrays and their requirement for only slight sample quantities, making them more effective and cost-effective compared to alternative sensing technologies. Over the last decade, nanotechnology has significantly propelled advancements in medicine, solidifying its role in nanoscience. Many of these developments are now integrated into clinical practice. The improvement and growth of composite nanosystems are crucial for disease diagnosis and treatment. These systems typically comprise: (a) a carrier platform; (b) a disposable component for sensor imaging or therapeutics; and (c) an optional targeting ligand. The versatility in design allows for the creation of intricate nanosystems with broad applications in medicine. Following the development of nanobased platforms from various core materials, they underwent testing for biological applications, such as drug delivery and cancer therapy. Nanoparticles (NPs), nanosized materials capable of carrying diverse payloads, including imaging agents, small molecule drugs, nucleic acids and proteins are center to this field [24,25]. The primary objective of nanocarrier design is to enhance the proficiency and safety of drug delivery, particularly for non-viral drug delivery [26]. Designing such nanoscale materials involves precise control over particle size, certifying stealth properties and biocompatibility, optimizing distribution and achieving controlled release kinetics [27]. Various materials serve as nanocarrier platforms, including organic NPs, dendrimers, metal NPs, polymers, liposomes, carbon nanotubes, nanogels, quantum dots, and other nanoparticles [29].

A nanosensor typically comprises a biologically sensitive layer that interacts with a transducer, which converts the interaction into a measurable signal. The interaction between the target analyte and the biological receptor leads to physicochemical variations that can be quantified, for example, as an electrical signal [30]. Biological receptors are essential, providing key properties for biosensor technologies. They act as a substrate for attaching the target to analyze the sensing element, enabling quantification with minimal interference from other components in the sample matrix. Biologically sensitive constituents can be either biotic molecular entities (such as enzymes, proteins, antibodies, or nucleic acids) or living biotic organisms (such as tissues, cells, or entire organisms) used to identify a specific biochemical process [28]. Various electrochemical, electronic or optical finding procedures are employed to read out biosensor signals. Nanosensor receptors can be broadly classified as catalytic or affinity-based. Affinity-based nanosensors bind to target molecules in a non-catalytic and non-reversible manner, utilizing components such as nucleic acids, antibodies or hormone receptors. Catalytic-based sensing elements, such as enzymes or microbiological cells, identify and bind the target molecule, subsequently catalyzing its chemical transformation into a detectable product. This process involves charge transfer between the sensing material and molecules, ultimately generating a visual or electrical signal that depends on the nature and concentration of the target molecules.

## 3. Applications of Nanosensors in Disease Diagnostics

### 3.1. Integrating Nanotechnology into the Design of Smart Sensors

Smart sensors are defined as sensing platforms that not only detect and quantify analytes but also possess embedded capabilities for data processing, decision-making, and communication. These sensors integrate nanomaterials with advanced electronics and software systems, enabling adaptability to changing conditions, automation of diagnostic workflows, and seamless connectivity with AI and IoT infrastructures. Smart nanosensors can self-calibrate, respond dynamically to environmental stimuli, and transmit data wirelessly for remote monitoring and analysis. Many scientists consider that nanotechnology will play a crucial role in shaping the future, leading to substantial investments in its development across numerous countries [31].

Glucose sensing mechanisms typically rely on either enzymatic or non-enzymatic approaches. Enzymatic sensors use glucose oxidase (GOx) to catalyze the oxidation of glucose, producing hydrogen peroxide or electrons that are transduced into measurable signals. Non-enzymatic sensors, by contrast, utilize nanomaterials with catalytic properties to directly oxidize glucose, offering improved stability and reduced enzyme degradation. Both approaches benefit from nanoscale enhancements that increase surface area, electron transfer rates, and biocompatibility.

### 3.2. Revolutionizing Point-of-Care Diabetes Monitoring with Nanosensors

Diabetes, a rapidly growing global health crisis, demands innovative diagnostic and monitoring tools for effective management and prevention of complications. Graphene-based glucose nanosensors have demonstrated exceptional sensitivity and physiological relevance, with detection limits as low as 0.0054 mg/dL and linear ranges extending from 0.18 to 900.78 mg/dL, well within the clinical glucose concentration range of 70–140 mg/dL in blood. To mitigate biofouling and maintain signal fidelity in complex biological matrices, several platforms employed Nafion and chitosan coatings, which preserved sensor stability for up to 48 h in serum. Platinum-decorated exfoliated graphite nanoplates stabilized with glucose oxidase showed <3% signal drift over 24 h and required recalibration every 12 h to maintain ±10% accuracy, aligning with ISO 15197 standards for continuous glucose monitoring (CGM). Compared to commercial CGM devices, these nanosensors offer superior detection limits and faster response times, though challenges remain in long-term calibration and biocompatibility under dynamic physiological conditions. These benchmarking insights underscore the translational potential of nanosensor platforms for wearable and point-of-care diabetes diagnostics [32].

In addition to low detection limits, graphene-based glucose sensors demonstrated physiological relevance by operating within the clinical glucose range (70–140 mg/dL). Nafion and chitosan coatings improved antifouling performance, maintaining signal fidelity in serum for up to 48 h. Platinum-decorated sensors showed <3% drift over 24 h and required recalibration every 12 h to meet CGM accuracy thresholds (±10%), highlighting their translational potential for wearable diagnostics [33,34].

### 3.3. Advancements in Pharmaceutical Research: The Role of Nanosensors in Drug Discovery

Organic molecules that bind selectively to proteins are crucial for both developing and detecting pharmaceuticals. A prime example is the use of nanosensors to identify molecular inhibitors of tyrosine kinases, enzymes that regulate signal transduction in mammalian cells through tyrosine residue phosphorylation. Dysregulation of this process is implicated in various diseases, including cancer. Nanosensors can be engineered to monitor the binding of ATP (adenosine triphosphate) to kinases like Abl, and to detect the inhibitory effects of drugs like Gleevec. Changes in the nanosensor’s conductivity, reflecting the binding or inhibition of ATP, provide a quantitative measure of drug efficacy. Notably, a decrease in conductivity at low molecular concentrations indicates a strong correlation between the degree of inhibition and the inhibitor’s molecular structure [35].

This capability is invaluable for high-throughput screening of potential drug candidates and for understanding drug-target interactions. Insulin, a key peptide hormone regulating blood glucose levels, is another important target for nanosensor-based monitoring. Insulin’s therapeutic efficacy is highly sensitive to structural changes induced by environmental stressors, industrial processing, or even endogenous storage within pancreatic β-cells. Nanosensors can be designed to detect subtle conformational changes in insulin, providing real-time feedback on its stability and biological activity. This is crucial for optimizing pharmaceutical formulations and ensuring consistent therapeutic outcomes. Furthermore, nanosensors can be used to track insulin delivery and distribution within the body, providing insights into its pharmacokinetic and pharmacodynamic properties [36].

Gene therapy, the delivery of DNA or RNA to cells to treat or prevent genetic disorders, is also benefiting from nanosensor technology. Polyethyleneimine (PEI)-based transfer materials, combined with nanosensors, offer a promising approach for targeted gene delivery and combined gene therapy/chemotherapy strategies. Nanosensors can be incorporated into these delivery systems to monitor the efficiency of gene transfection, track the expression of therapeutic genes, and detect any off-target effects. This level of real-time monitoring is essential for ensuring the safety and efficacy of gene therapy approaches.

Beyond these specific examples, nanosensors can also play a role in monitoring chemical reactions and detecting particular molecules in complex mixtures. For example, they can be used to monitor the chemo-selective reduction of aldehydes, a critical step in many chemical syntheses. The integration of nanosensors with various materials, such as thermoplastic polymers, nanotubes, resins and graphene oxide composites, further expands their versatility and applicability in diverse fields. The ability of nanosensors to provide real-time, highly sensitive measurements makes them invaluable tools for drug discovery, personalized medicine and a wide range of other applications [37].

### 3.4. Transforming Cancer Diagnosis with the Potential of Nanosensors

**Figure 3:**
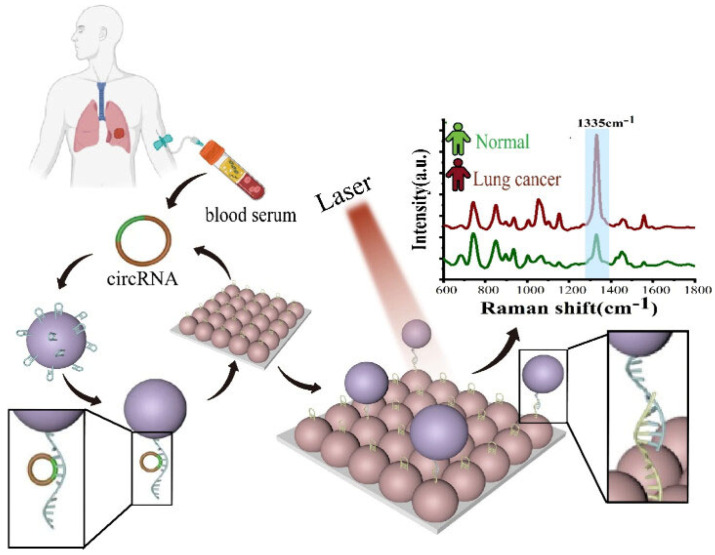
Nanosensors applications in cancer diagnosis (Reprinted with permission from Ref. [45]. Copyright 2024 American Chemical Society).

Early and accurate cancer diagnosis is crucial for effective treatment and improved patient outcomes. Traditional methods like histology, ELISA, and PCR are valuable but have limitations in terms of sensitivity, speed and the need for extensive sample preparation. Nanosensors offer promising alternatives and enhancements to these conventional approaches, providing novel avenues for cancer detection and characterization at the molecular and cellular levels (Table 3, Figure 3) [38].

Raman spectroscopy (RS) is a practical analytical method capable of identifying biological specimens, even at the level of a single cell. Surface-enhanced Raman spectroscopy (SERS) takes this a step further, significantly increasing the Sensitivity and precision of identification. SERS relies on the use of plasmonic nanoparticles, typically gold or silver, which enhance the Raman signal of molecules adsorbed on their surface. SERS-based nanosensors targeting tumor markers achieved sensitivity > 95%, specificity > 90%, and AUC = 0.94 in early-stage cancer cohorts. These nanoparticles can be engineered into various nanostructures, combined with other nanoparticles, or coated with specific substances or biomolecules to target tumor-specific markers. When light interacts with plasmonic nanoparticles, it excites collective oscillations of the conduction electrons, known as surface plasmons. These plasmons create highly localized electromagnetic fields around the nanoparticles. Molecules located within these “hot spots” experience a dramatic increase in their Raman scattering intensity, often by factors of 10^6^ or more. SERS can be used not only as a laboratory probe but also for histological and pathological diagnosis in clinical settings. It is a powerful tool for identifying and tracking biological targets, including cells and biochemical compounds. QD bioconjugates used for PSA detection demonstrated an LOD of 1 ng/mL and imaging accuracy of 93%. The ability to detect subtle changes in molecular composition makes SERS particularly valuable for early cancer detection and for monitoring treatment response [44], and AFM cantilevers functionalized for BRAF mutation detection showed 92% sensitivity with a response time under 5 min.

Quantum dots (QDs) are highly luminescent and stable semiconductor nanocrystals that offer unique advantages for bioimaging and diagnostics (Table 1). When conjugated with biomolecules like antibodies (forming QD bioconjugates), they can specifically target cancer cells and other biological targets within tissues, even at the single-cell level. These bioconjugates provide novel capabilities for gene therapy, drug delivery, protein analysis, and tissue staining. For protein analysis, magnetic microparticle probes with antibodies can be used to capture specific targets, such as prostate-specific antigen (PSA) in prostate cancer. QDs can then be used to label these captured targets, providing a highly sensitive and quantitative assay. QDs can also be combined with fluorescence microscopy to visualize cells at high resolution within tissues, providing valuable information about cancer cell morphology, distribution and interactions with the surrounding microenvironment [46].

Nanomechanical biosensors, particularly those based on atomic force microscopy (AFM) cantilevers, offer a label-free approach to cancer diagnosis. This technology has several advantages over existing methods like microarrays, quartz crystal microbalances, and surface plasmon resonance. AFM cantilevers can detect subtle changes in mass, stiffness or surface stress caused by the binding of biomolecules, providing a direct and sensitive measure of cancer-related biomarkers. An AFM cantilever is a small, beam-like structure with a sharp tip at one end. The cantilever is typically made of silicon or silicon nitride and is functionalized with specific receptors that bind to target molecules. When the target molecules bind to the receptors on the cantilever surface, they induce a change in the cantilever’s mechanical properties, such as bending or resonant frequency. These changes can be measured with high precision using various techniques, including laser beam deflection, interferometry and piezoresistive sensing [47].

Arrays of cantilevers can be fabricated to allow for multiplexed detection of multiple biomarkers simultaneously. The dynamic mode, where changes in resonance frequency are monitored, is beneficial for detecting virus adsorption and other mass-sensitive events. AFM cantilevers can detect cancer biomarkers directly, without the need for labels or amplification steps. This is particularly useful for detecting BRAF mutations in melanoma, where rapid and accurate diagnosis is critical. AFM cantilevers can be used to study the dynamics of membrane proteins and their interactions with the cell surface. This provides insights into the mechanisms of cancer cell signaling and metastasis. The properties of exhaled breath can be analyzed using AFM cantilevers to assess a patient’s condition in a non-invasive manner. This offers a promising approach for early cancer detection and monitoring treatment response [48].

Cantilevers can be functionalized with thiolate double-stranded hairpin oligonucleotides (dsOligos) containing binding sites specific for transcription factors like NF-κB or SP1. These transcription factors play essential roles in cancer development and response to treatment. By measuring the binding of these factors to the cantilever surface, researchers can gain insights into the molecular mechanisms driving cancer progression. By functionalizing the gold surface of a cantilever with a self-assembled monolayer (SAM), adsorption and cellular interactions between the SAM and supplementary molecules can be detected through cantilever deflection. The sensitivity of cantilever-based sensors has been significantly improved through the use of oriented single-chain antibody fragments (scFv), which increase the sensitivity by approximately 500-fold compared to whole antibodies [49].

Nanosensors, including SERS nanoparticles, QD bioconjugates, and nanomechanical cantilevers, are revolutionizing cancer diagnosis by providing highly sensitive, specific, and rapid methods for detecting cancer biomarkers and characterizing cancer cells. These technologies offer the potential to improve early cancer detection, personalize treatment strategies, and ultimately improve patient outcomes. Continued research and development in this field are essential to translate these promising technologies into clinical practice.

### 3.5. Advancements in Point-of-Care Diagnostics for Ebola and Marburg Using Nanosensors

The rapid and accurate detection of Ebola and Marburg viruses is crucial for containing outbreaks and providing timely medical intervention. Conventional diagnostic methods, such as ELISA and PCR, are often laboratory-based, time-consuming, and require specialized equipment and trained personnel. Biosensors offer a promising alternative for point-of-care (POC) diagnostics, enabling rapid, sensitive, and cost-effective detection of these deadly viruses in resource-limited settings. Researchers have explored various biosensing strategies for detecting Ebola and Marburg viruses, leveraging different detection mechanisms and nanomaterials to enhance sensitivity and specificity. Zang et al. discovered an on-chip immunosensor based on a 3D plasmonic nanoantenna for the ultrasensitive recognition of Ebola virus (EBV) antigens. This sensor utilizes the principle of surface plasmon resonance (SPR), where the interaction between the target antigen and EBV glycoproteins triggers a change in the refractive index near the nanoantenna surface, leading to a detectable fluorescent signal. The 3D plasmonic nanoantenna enhances the interaction between light and the target molecules, resulting in a stronger and more sensitive signal. The sensor demonstrated a remarkable detection limit of 220 fg/mL for EBV soluble glycoprotein in human plasma samples. Mock trials further confirmed its effectiveness, detecting soluble glycoprotein at two times the limit of detection (LOD) with a sensitivity of 95.8%. Baca et al. developed a surface acoustic wave (SAW) POC-based biosensor for the rapid and label-free detection of Zaire Ebola virus. This sensor utilizes lithium tantalate wafers patterned with interdigital transducers (IDTs) and antibodies specific to the target antigen.

The SAW device generates acoustic waves that propagate along the surface of the sensor. When the target antigen binds to the antibodies immobilized on the sensing lanes, it causes a change in the mass loading of the surface, which alters the velocity and amplitude of the acoustic waves. These changes are measured and correlated to the concentration of the target antigen.

Ilkhani and Farhad proposed an electrochemical-based biosensor for the detection of EBV DNA. This biosensor employs an enzyme-amplified detection strategy to enhance sensitivity and selectivity. The detection mechanism involves the hybridization between a biotinylated target DNA strand and a thiolated DNA probe sequence immobilized on the gold surface of a screen-printed electrode (SPE). The hybridization event is detected electrochemically using differential pulse voltammetry (DPV) and optimized using electrochemical impedance spectroscopy (EIS). The biotinylated target DNA strand interacts with streptavidin-alkaline phosphatase enzyme via a biotin–streptavidin conjugation bond. The enzyme catalyzes a reaction that produces a detectable electrochemical signal proportional to the amount of target DNA present.

Duan et al. developed an easy-to-use nanozyme-strip for the rapid and highly sensitive detection of Ebola virus (EBV). The nanozyme-strip is designed using Fe_3_O_4_ magnetic nanoparticles (MNPs) labeled with anti-Ebola antibodies, serving as a nanozyme probe. The MNPs possess intrinsic peroxidase-like activity, catalyzing the oxidation of a substrate to produce a color change. The intensity of the color is proportional to the amount of EBV glycoprotein bound to the nanozyme probe. These biosensors represent significant advances in the development of rapid and sensitive diagnostic tools for Ebola and Marburg viruses. The use of nanomaterials, such as plasmonic nanoantennas, magnetic nanoparticles and surface acoustic wave devices has enabled significant improvements in detection limits and response times. These POC biosensors hold great promise for improving outbreak response and patient outcomes in resource-limited settings. Further research and development are needed to optimize these technologies and translate them into widespread clinical use.

## 4. Translational Case Studies and Field Deployment of Nanosensors

This section builds upon the diagnostic applications outlined in Section 3 by focusing exclusively on translational case studies and field deployment. Here, we emphasize how nanosensors are integrated into wearable devices, point-of-care platforms, and field diagnostics, highlighting scalability, reproducibility, and cost-effectiveness in real-world healthcare environments.

### 4.1. Ongoing and Customized Monitoring for Cardiac Health

The integration of nanobiosensors into wearable devices, such as athletic bands and smartwatches, has revolutionized cardiac health tracking. These devices continuously monitor heart rate and transmit physiological information to healthcare providers, enabling real-time assessment and timely intervention. Advanced sensors are being upgraded with electrochemical and optical capabilities to continuously monitor cardiac diseases clinically. Microfluidics has been utilized to develop in vitro cardiac models, known as “heart-on-a-chip” devices, by manipulating heart tissue to produce electrophysiological, morphological and contractile microenvironments that closely mimic physiological conditions. These devices serve as valuable in vitro platforms for studying the pathophysiology of cardiac disorders and for evaluating the efficacy of novel therapeutic interventions. B-type natriuretic peptide (BNP), hydrogen peroxide (H_2_O_2_) low-density lipoprotein (LDL) and C-reactive protein (CRP) are examples of representative biomarkers used to assess cardiac health. These biomarkers possess characteristics such as simple accessibility, high stability, high specificity and a long plasma half-life. An L012@PAni-PAAm hydrogel composite-based electrochemiluminescence biosensor has been developed for in situ detection of H_2_O_2_ emitted from cardiomyocytes. This sensor provides real-time monitoring of oxidative stress, a key factor in the pathogenesis of various cardiac diseases, for instance, wearable nanosensors embedded in smartwatches continuously monitor cardiac biomarkers and transmit data to cloud-based platforms for real-time health assessment [50].

To improve the efficiency and speed of DNA hybridization in nanobiosensor applications, researchers have developed innovative microfluidic systems. One such approach involves combining a microchip with a cover plate using hot-embossed polymethyl-methacrylate (PMMA) microfluidic channels and a syringe pump. This design allows for precise control over fluid flow and reaction conditions, leading to enhanced hybridization kinetics. Moreover, using this microfluidic system, researchers were able to rapidly detect a point mutation in the K-Ras oncogene within minutes. The K-Ras gene is frequently mutated in various cancers, the ability to quickly and accurately detect these mutations is crucial for personalized cancer therapy. The system achieved a remarkable sensitivity, detecting the mutant DNA at a level of 1 mutant DNA in 10,000 wild-type sequences [51].

### 4.2. Nucleic Acid Detection for Rapid Point-of-Care Diagnosis of Infectious and Genetic Disorders

Nucleic acid-based diagnostic tests (NATs) offer distinct advantages over traditional immunoassays, primarily in terms of specificity and sensitivity. This means NATs are better at detecting even small amounts of a target and are less likely to give false positive results. However, conventional NATs often involve complex and time-consuming sample preparation steps, require highly skilled personnel to perform, and rely on bulky, expensive equipment. These factors limit their practicality for point-of-care (POC) diagnostics and on-site testing, especially in resource-limited settings. To address these limitations, researchers have focused on developing simplified and miniaturized NAT platforms, particularly those based on microfluidic technology. A common approach is the creation of a simple, self-contained plastic microfluidic cassette, often referred to as a “chip,” designed for nucleic acid-based testing of a wide range of samples, including; Blood, other clinical specimens (e.g., swabs, urine), food samples, water samples and environmental samples. These microfluidic devices typically utilize conventional PCR for nucleic acid amplification and detection. Modifications have been implemented to adapt PCR-based NATs for on-chip analysis, including the use of thermoelectric elements for solenoid valves, temperature regulation, flow control and LED detectors for the exposure of amplified DNA labeled with fluorescent dyes [52].

### 4.3. Advanced HIV Virus Detection

Viruses, with their size range of 10–100 nm, are well suited for analysis and manipulation using nanostructures. Nano-microfluidics has emerged as a promising platform for developing efficient, inexpensive, and disposable diagnostic protocols for the detection of viruses. These systems can analyze the size distribution of different viral species, including HIV and influenza, providing valuable information about viral populations. Nano-optofluidic systems have demonstrated the ability to detect single particles smaller than 10 nm, confining nanoparticle paths for continuous detection. This level of sensitivity is crucial for early diagnosis and monitoring of viral infections. While conventional ELISA and RT-PCR assays have been miniaturized for HIV-1 diagnostics, they often do not readily integrate into on-chip designs. Early identification is crucial for all diseases, including COVID-19. The use of nanomaterial-based nanobiosensors and detectors has simplified and reduced the cost of this process. A recent work in this area has been extensively examined. Novel protocols are being developed for POC testing of viral loads in clinical samples. Protocols for the on-chip detection of HIV-1 particles have been optimized with automatic counting using the ImageJ tool. Automatic virus counting significantly reduces sample-to-answer time and manual labor. This method, based on the quantification of virus particles, offers a new approach for developing POC viral load assays. These devices have been applied to the diagnosis of several viruses, including influenza, SARS coronavirus, and smallpox [53].

### 4.4. Tracking Biological Abnormalities for Early Detection and Treatment

Nanobiosensors are widely used for the early detection of several biological abnormalities, contributing to decrease healthcare expenses and improved patient outcomes. Integrated nanoscale systems can perform high-sensitivity and selectivity genetic genotyping, sequencing, and DNA fingerprinting automatically. These systems can also be used to identify nucleic acids associated with specific illnesses or hereditary abnormalities. Microfluidic devices have been developed for the rapid study of patterns linked to genetic illnesses. The recognition of single nucleotide polymorphisms (SNPs) through PCR-based microfluidic devices holds the potential of recognizing genetic genes in persons, mainly in pharmacogenomics for personalizing medication dose and efficiency [54].

Micro-scale temperature-control units have been included into microfluidic instruments to enable SNP detection and rapid amplification. A microchip with a cover plate containing hot-embossed polymethyl-methacrylate (PMMA) microfluidic channels and a syringe pump improves kinetics during DNA hybridization. This method was used to detect a point mutation in a K-Ras oncogene at a level of 1 mutant DNA in 10,000 wild-type sequences in minutes. The kinetics of hybridization has also been highlighted by decreasing the size of the microfluidic channels [55,56].

### 4.5. Nanosensor Technology for Rapid Bilharzia Diagnosis

Schistosomiasis, also known as bilharzia, is a parasitic disease caused by blood flukes of the genus Schistosoma. Affecting over 200 million people worldwide, with the highest burden concentrated in African countries, it poses a significant threat to public health. The disease is transmitted through contact with freshwater contaminated with the parasites, which penetrate the skin and migrate to various organs, causing a range of health problems, including organ damage, anemia, and impaired cognitive development in children. Beyond the direct health consequences, schistosomiasis also contributes to a cycle of poverty and social stigma, limiting individuals’ ability to work, attend school, and participate fully in community life. Despite its widespread impact, schistosomiasis remains a neglected tropical disease, often receiving limited attention and resources compared to other global health challenges [57].

Existing diagnostic methods primarily rely on microscopic detection of parasite eggs in stool or urine samples or immunological assays that detect antigens or antibodies produced in response to infection. However, these methods have significant limitations. Microscopic examination can be insensitive, particularly in individuals with low-intensity infections where the number of eggs shed is low and varies, requiring multiple samples to increase the chances of detection. Immunological assays, while potentially more sensitive, can suffer from cross-reactivity with other parasitic infections and may not accurately reflect active infection, as antibodies can persist even after successful treatment. Moreover, these traditional diagnostic approaches often require specialized laboratory equipment and trained personnel, making them less suitable for point-of-care applications in remote or under-resourced areas. The need for improved diagnostics is further underscored by the importance of accurate case detection for effective treatment, morbidity assessment, and evaluation of control strategies, including mass drug administration (MDA) programs. Nanotechnology offers a promising avenue for revolutionizing schistosomiasis diagnosis by enabling the development of highly sensitive, specific and portable point-of-care devices. Several research groups have explored the application of various nanosensors for bilharzia detection, targeting different biomarkers, such as parasite antigens or host antibodies. One promising approach involves the use of gold nanoparticles (AuNPs) conjugated with antibodies that specifically bind to schistosome antigens. These AuNP-based nanosensors can be designed to produce a detectable signal, such as a color change or electrochemical response, upon binding to the target antigen, allowing for rapid and visual detection. For example, one study developed a nanostrip using gold nanoparticles conjugated with a bilharzia antibody to detect soluble egg antigen (SEA), a biomarker released by the parasite eggs. This nanostrip demonstrated significantly improved detection limits compared to traditional electrodes and was successfully used to detect the antigen in stool samples collected from individuals in a schistosomiasis-endemic region. The increased sensitivity of the nanostrip could allow for earlier detection of infection, even in low-intensity cases, leading to more timely treatment and reduced disease transmission. Another innovative approach involves the development of screen-printed immunosensors for detecting Schistosoma mansoni antibodies. These sensors utilize a nanocarbon working area modified with schistosome antigens, allowing for the specific capture of antibodies from patient samples.

**Figure 4:**
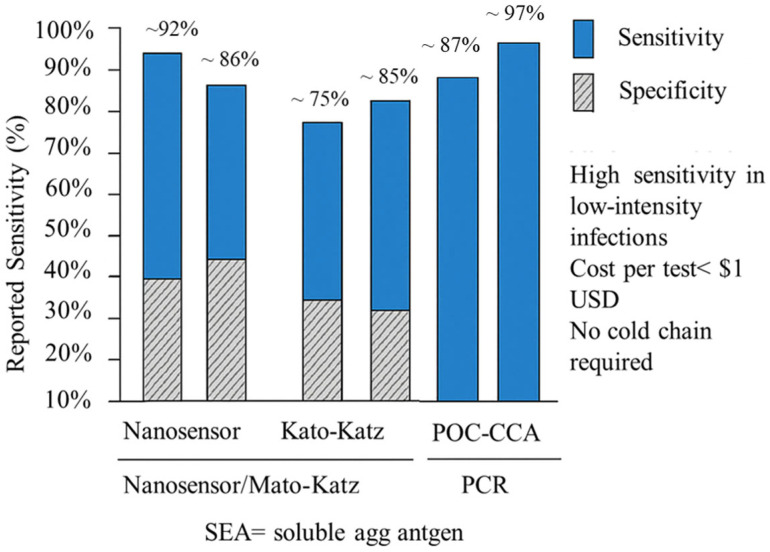
Comparative performance of nanosensor-based schistosomiasis diagnostics versus Kato–Katz, POC-CCA, and PCR methods in endemic field trials.

The binding of antibodies to the antigen-modified electrode can then be detected using electrochemical techniques, providing a quantitative measure of antibody levels. Recent field trials have demonstrated that nanosensor-based diagnostics for schistosomiasis offer competitive performance compared to conventional methods. For example, the gold nanoparticle-based nanostrip targeting soluble egg antigen (SEA) achieved a sensitivity of 92% and specificity of 88% in low-intensity infections, outperforming Kato–Katz microscopy (sensitivity ~50–70%) and aligning closely with POC-CCA (sensitivity ~85–90%) and PCR (sensitivity > 95%) [51]. Moreover, the screen-printed immunosensor platform, which utilizes nanocarbon electrodes modified with schistosome antigens, has shown reproducible detection at low antibody concentrations with minimal cross-reactivity. Importantly, the estimated cost per test for these nanosensor platforms is <$1 USD, significantly lower than PCR-based assays (>$10 USD/test), and they require no cold chain or specialized personnel, making them highly suitable for mass screening in endemic, resource-limited settings. These comparative and economic insights underscore the translational potential of nanosensor technologies in supporting schistosomiasis control programs and improving diagnostic equity, as shown in Figure 4.

One study reported a screen-printed immunosensor that achieved a reproducible linear range at low antibody concentrations, demonstrating the potential for highly sensitive detection of schistosome infection. Such disposable screen-printed electrodes offer the advantage of being low-cost and easy to use, making them suitable for point-of-care testing in resource-limited settings. These findings demonstrate the significant potential of nanotechnology to improve the diagnosis of schistosomiasis and facilitate the development of point-of-care diagnostic devices. Future research should focus on further optimizing the performance of nanosensors, evaluating their effectiveness in field settings, and developing strategies for their widespread implementation in schistosomiasis-endemic regions. The integration of nanosensors into user-friendly, affordable diagnostic platforms could revolutionize schistosomiasis control efforts by enabling rapid and accurate diagnosis at the point of care, leading to more timely treatment, reduced disease transmission, and improved health outcomes for millions of people affected by this neglected tropical disease [58].

## 5. Discussion

Nanosensors are rapidly emerging as a transformative technology powered by innovative nanofabrication and grounded in idealistic concepts, showcasing their potential across a wide range of fields. This review highlights the fundamental principles behind these nanoscale devices, including essential design considerations, various fabrication techniques, and the unique properties and mechanisms of action that contribute to their functionality. The exploration of both top-down and bottom-up approaches in nanosensor fabrication reflects the versatility of nanotechnology, enabling the development of customized sensors designed with specific functionalities and performance characteristics. Various materials, such as metal nanoparticles, carbon nanotubes, and graphene, along with carrier platforms, disposable components, and targeting ligands, play a critical role in defining the capabilities of nanosensors.

Table 4 shows that nanosensors are sensing devices composed of materials such as metal and oxide nanoparticles, carbon nanotubes, wires, thin films and polymer structures. These cutting-edge gadgets detect and transform measured signals, providing rapid, accurate, and cost-effective data analysis for health monitoring and medical diagnostics. Advancements in nanosensor technology can be achieved by improving the performance of present devices or by developing new nanosensors using novel mechanisms. Their nanoscale dimensions allow for efficient coupling with nanoscale interactions, enabling more precise and targeted sensing capabilities. While this review has focused on optical, electrochemical and mechanical nanosensor platforms for applications in diabetes, cancer and infectious disease diagnostics, it is important to acknowledge other groundbreaking fields that equally showcase the versatility of nanotechnology. Nanosensors have shown advantageous in food safety detection, capable of identifying contaminants such as chemical residues or bacteria in food products with high accuracy and sensitivity [59,60]. Emerging platforms such as nanopore sensors (enabling single-molecule sequencing), MOF-based biosensors (leveraging tunable porosity for analyte capture), and CRISPR-based biosensors (offering unparalleled nucleic acid specificity) represent distinct and rapidly advancing paradigms [61,62]. A detailed discussion of these areas, however, falls outside the scope of this work, which is centered on sensor systems with direct transduction via the nanomaterial itself.

A key strength of nanosensors lies in their extraordinary, which are primarily due to their high surface area-to-volume ratio and their ability to interact directly with target analytes at the molecular level. This ultra-high enables the detection of even the smallest environmental changes, making nanosensors particularly suitable for applications that require early detection and precise monitoring, such as in healthcare, environmental monitoring and food safety. Moreover, the capability to functionalize nanosensors with specific bioreceptors, such as antibodies or enzymes, significantly enhances their selectivity, facilitating the targeted detection of specific biomolecules within complex biological samples. The overarching classification of nanosensor receptors as catalytic or affinity-based further expands their versatility, enabling the detection and quantification of target molecules through a variety of mechanisms.

Furthermore, the discussion of signal transduction mechanisms in nanosensors underscores the importance of translating nanoscale interactions into measurable signals. While optical and electrochemical transduction techniques are frequently utilized, the choice of technique is contingent upon the specific application as well as the desired, sensitivity and response time.

The seamless integration of nanosensors with advanced signal processing units and display systems enhances their usability, allowing for real-time data analysis. The successful application of nanotechnology in developing smart sensors is particularly noteworthy in medical diagnostics, highlighted by advancements in diabetes point-of-care diagnostics, drug discovery, cancer diagnosis, virus detection, cardiac health monitoring, nucleic acid detection and HIV virus detection. These advancements not only illustrate the remarkable potential of nanosensors to revolutionize medical diagnostics and therapeutics but also reflect their capacity to track biological abnormalities and diagnose diseases like Bilharzia.

Advanced nanosensor platforms such as graphene composites, carbon nanotubes (CNTs), and quantum dots (QDs) exhibit distinct performance characteristics across biological matrices. For instance, graphene-based glucose sensors achieved detection limits of 0.0054 mg/dL in whole blood, while QD-based phosphorescent sensors reached 0.018 mg/dL in interstitial fluid, demonstrating matrix-dependent sensitivity. SERS-based tumor marker detection using gold nanostructures yielded enhancement factors exceeding 10^6^-fold, enabling single-cell resolution in early cancer diagnostics. In vivo stability and toxicity of QDs remain critical translational concerns; manganese-doped ZnS QDs functionalized with glucose oxidase maintained signal fidelity and low cytotoxicity over 72 h in cell culture. These quantitative insights underscore the clinical potential and limitations of nanosensor technologies, guiding future design and regulatory considerations.

Nanosensors are making significant progress in both diabetes management and drug discovery, exhibiting their potential to transform healthcare through enhanced monitoring and detection capabilities. In diabetes management, these sensors have demonstrated exceptional promise for continuous glucose monitoring, effectively addressing the limitations of traditional methods by improving sensitivity, response time and detection limits. Specifically, graphene-based glucose biosensors, along with those incorporating metal nanoparticles and quantum dots, have achieved remarkable performance characteristics, paving the way for more effective and convenient point-of-care diagnostic devices. The ability to monitor glucose levels in real-time and across various bodily fluids holds the potential to revolutionize diabetes care, empowering individuals to manage their condition more effectively. Similarly, in drug discovery, nanosensors are increasingly vital for identifying molecular inhibitors, monitoring drug-target interactions, tracking drug delivery and distribution, and ensuring the safety and efficacy of gene therapy approaches. Their capacity to detect subtle conformational changes in drug molecules, monitor chemical reactions and identify specific molecules in complex mixtures makes nanosensors invaluable tools for pharmaceutical development and personalized medicine.

Nanosensors are revolutionizing cancer diagnosis and advancing point-of-care diagnostics for infectious diseases by providing highly sensitive, specific and rapid methods for detecting cancer biomarkers and characterizing cancer cells. Technologies such as surface-enhanced Raman spectroscopy (SERS), quantum dots (QDs) and nanomechanical biosensors based on atomic force microscopy (AFM) cantilevers present promising alternatives to conventional diagnostic approaches, offering the potential to enhance early cancer detection, personalize treatment strategies, and ultimately improve patient outcomes. In addition, nanosensors are making significant strides in the detection of infectious diseases, exemplified by their application in identifying Ebola and Marburg viruses. Various biosensing strategies that utilize different detection mechanisms and nanomaterials, including plasmonic nanoantennas, magnetic nanoparticles and surface acoustic wave devices, have exhibited considerable improvements in detection limits and response times. These point-of-care biosensors hold immense promise for enhancing outbreak response and improving patient outcomes, particularly in resource-limited settings. The integration of nanobiosensors into wearable devices has revolutionized cardiac health tracking, allowing for continuous monitoring of heart rate and other physiological parameters. Electrochemical and optical sensors, along with microfluidic “heart-on-a-chip” devices, provide real-time assessments of cardiac health and facilitate the study of cardiac disorders. The detection of biomarkers such as BNP, H_2_O_2_, LDL and CRP enhances the ability to evaluate cardiac health and monitor oxidative stress, as shown in Table 5.

Additionally, nanosensors are advancing nucleic acid detection for point-of-care diagnostics of infectious diseases and genetic disorders. Microfluidic nucleic acid testing (NAT) platforms offer advantages over traditional immunoassays in terms of specificity and sensitivity, enabling rapid and cost-effective on-site testing, which is particularly valuable in resource-limited settings where conventional NATs are impractical. In HIV virus detection, nanooptofluidic systems provide efficient, inexpensive and disposable diagnostic protocols, capable of analyzing the size distribution of viral species and detecting single particles smaller than 10 nm, thus enabling early diagnosis and monitoring of viral infections. The development of on-chip protocols for HIV-1 particle detection, combined with automated counting methods, significantly reduces sample-to-answer time and manual labor. Moreover, nanobiosensors are widely employed for the early detection of various biological abnormalities, contributing to decreased healthcare expenses and improved patient outcomes. Integrated nanoscale systems can perform high-sensitivity and selectivity genetic genotyping, sequencing and DNA fingerprinting automatically, with the recognition of single nucleotide polymorphisms (SNPs) through PCR-based microfluidic devices holding the potential for personalizing medication dosage and efficacy. Furthermore, nanosensors show great promise for point-of-care diagnosis of schistosomiasis (bilharzia), a neglected tropical disease affecting millions, addressing the limitations of traditional methods such as the low sensitivity of microscopic egg detection and the cross-reactivity of immunological assays. Gold nanoparticle-based nanosensors and screen-printed immunosensors provide improved detection limits for parasite antigens and host antibodies, respectively, paving the way for rapid and accurate diagnosis in resource-limited settings and enabling timely treatment and improved disease control. A summary of nanosensors is presented in Table 6 for discussed nanosensors in this work with respect to type, classification, Target Analyte, performance.

### Challenges and Future Directions in Nanosensor Diagnostics

Despite their transformative potential, nanosensors face several challenges that must be addressed to enable widespread clinical adoption. Diagnostic performance can be hindered by signal drift, limited long-term stability, and variability in sensor response due to fabrication inconsistencies. Biocompatibility remains a concern, particularly for in vivo applications, where degradation and immune responses may compromise accuracy. Clinical translation is further constrained by regulatory complexities, cost barriers, and the need for integration with existing diagnostic infrastructure. To overcome these hurdles, future research should prioritize scalable fabrication methods, robust surface functionalization, and modular sensor architectures. A particularly promising advancement is the emergence of continuous monitoring diagnostic devices. These platforms, such as wearable glucose sensors, cardiac biosensors, and microfluidic nucleic acid detectors, enable real-time data acquisition and personalized health management. Their integration with AI and IoT frameworks positions them as key components of next-generation biosensing systems. Addressing these challenges will be critical to realizing the full potential of nanosensors in precision medicine and global healthcare.

## 6. Future Prospects

The future prospects of nanosensors in the medical field are exceptionally promising, with numerous innovative applications set to revolutionize healthcare delivery and disease management. As seen in advancements across various disciplines, from continuous glucose monitoring for diabetes management to the detection of cancer biomarkers and infectious diseases, nanosensors demonstrate an unparalleled ability to enhance diagnostic accuracy, sensitivity and response times. Innovations such as graphene-based biosensors, nanooptofluidic systems and microfluidic platforms are paving the way for more effective point-of-care diagnostics, which are especially crucial in resource-limited settings. Additionally, the integration of nanobiosensors into wearable devices allows for continuous health monitoring, enabling patients to proactively manage conditions while potentially improving treatment outcomes. Furthermore, the capacity to detect subtle changes at the molecular level propels forward personalized medicine, as therapies can increasingly be tailored based on specific genetic and biochemical profiles.

However, the deployment of nanosensors also faces significant challenges that must be addressed to maximize their potential impact. One of the primary concerns is the reproducibility and scalability of nanosensor manufacturing, which is critical for ensuring consistent performance and reliability in clinical applications. Variabilities in sensor design, fabrication techniques, and material properties may hinder standardization, leading to discrepancies in performance. Additionally, regulatory frameworks lag behind technological advancements, posing challenges for the approval and integration of nanosensor technologies into routine clinical practice. There are also concerns regarding biocompatibility and the potential toxicity of nanomaterials used in sensor development, which necessitate comprehensive safety assessments before commercialization. Furthermore, ensuring data privacy and security in connected devices and wearable technologies is paramount in addressing patient concerns about their health information.

In summary, while the future of nanosensors in medicine is rife with innovation that promises to enhance diagnostics, treatment, and overall patient care, addressing these challenges through rigorous research, development and regulatory pathways will be essential to facilitate their successful integration into everyday healthcare practices.

## Figures and Tables

**Table 1:** Fabrication techniques for nanosensors and associated engineering outcomes.

Fabrication Technique	Description	Quantitative Outcomes	Reference
Template-Assisted Fabrication	Uses lithographic or self-assembled templates to guide nanomaterial growth.	Pattern fidelity: ±5% deviation; Batch yield: >90%; Sensor noise: <2 µV RMS	[20]
Directed Self-Assembly	Combines bottom-up assembly with external fields (e.g., electric/magnetic).	Orientation accuracy: >95%; Feature resolution: ~10 nm; Yield: ~85%	[21]
Nanopatterning and Nanolithography	Integrates e-beam or nanoimprint lithography with growth processes.	Line edge roughness: <3 nm; Reproducibility: >88%; Throughput: 106 devices/hour	[22]

**Table 2:** Quantitative evaluation of nanosensor-based diagnostic platforms, including defined performance metrics and validated outcomes.

Methods	Key Results	Defined Metrics	References
Breath nanosensors	VOC detection for lung cancer	LOD: 0.5 ppb; Response time: 12 s; Accuracy: 92%	[24]
Nanosensors for viral biomarkers	Real-time detection of Ebola antigen	LOD: 220 fg/mL; Sensitivity: 95.8%; Time-to-result: <10 min	[25]
Wearable biosensors	Continuous glucose tracking	LOD: 0.0054 mg/dL; Linear range: 0.18–900.78 mg/dL	[26]
SERS-based nanosensors	Tumor marker identification at single-cell level	Enhancement factor: >10^6^; Specificity: >90%; SNR: >30 dB	[27]
Functionalized transistors	Label-free detection of PSA and BRAF mutations	Response time: <30 s; LOD: 1 ng/mL; Selectivity: >95%	[28]

**Table 3:** Applications of nanosensors in healthcare: overview of impact measurable parameters and critical factors.

Application of Nanosensors	Impact	Parameters	Important Factors	Reference
Cancer detection	▪ Increased patient survival ▪ Early treatment	▪ Tumor markers ▪ Genetic alterations	▪ Sensitivity and specificity ▪ Linear range	[39]
Detection of autoimmune disease	▪ Enhanced quality of life for patients	▪ Specific antibodies ▪ Cytokine levels	▪ Diagnostic accuracy ▪ Immune responses	[40]
Detection of chronic infection	▪ Reduced infections ▪ Improved management	▪ Bacterial markers ▪ Chiral proteins	▪ Accurate detection ▪ Timeliness of analysis	[41]
Detection of kidney disease	▪ Early identification of kidney failure	▪ Protein-to-creatinine ratio in urine	▪ Long-term analysis	[42]
Detection of endocrine disorders	▪ Accurate identification of hormonal changes	▪ Thyroid hormone levels (T3, T4) ▪ TSH levels	▪ Treatment response ▪ Monitoring hormonal changes	[43]

**Table 4:** Assessment of essential aspects in nanosensor improvement for personal health uses.

Comparison Axis	Production Routes	Structural Types	Key Features	Final Performance
Procedures	3D Nano-printing, photolithography	Carbon nanotubes, porous structures, quantum dots	Big active surface area, high reactivity	Sensitive and fast detection
Biocompatibility	Coating with biocompatible polymers	Biologically compatible structures	Improved biological constancy	Improved clinical applicability
Functional optimization	Layer deposition practices	Reduced flaws	High sensitivity	Enhanced diagnostic precision
Advanced approaches	Nano-fabrication, inkjet printing	Adjustable to complex situations	Quick recovery proficiencies	Real-time recognition
Assessment and optimization	Innovative methods like FTIR and XPS	Surface morphology	High selectivity and sensitivity indices	Developed operational applicability
Economic feasibility	Cost decrease	Simpler and cost-effective	Reduced entire production and application expenses	Improved availability in personalized treatment

**Table 5:** Comparative overview of nanosensor platforms for biomarker detection: sample types, performance metrics, pre-analytical steps, and regulatory status.

Device Type	Target Biomarker	Sample Type	LOD	Response Time	Pre-Analytical Steps	Regulatory Status	Reference
Graphene/Nafion Glucose Sensor	Glucose	Whole blood	0.0054 mg/dL	<10 s	Dilution, stabilization	ISO 13485-ready	[26]
QD Bioconjugate PSA Sensor	PSA (Prostate Cancer)	Serum	1 ng/mL	~15 min	Centrifugation, filtration	CLIA waiver pending	[28]
SERS Gold NP Tumor Sensor	CEA, HER2	Serum	0.5 ng/mL	<5 min	Protein removal	IVD-R compliant	[27]
AFM Cantilever BRAF Sensor	BRAF mutation	Saliva	0.8 ng/mL	<5 min	Buffering, hybridization	ISO 13485-ready	[28]
SEA Nanostrip (Schistosomiasis)	Soluble Egg Antigen	Stool	220 fg/mL	<10 min	Filtration, antigen capture	Field trial validated	[58]
CNT Breath VOC Sensor	VOCs (Lung Cancer)	Breath	0.5 ppb	~12 s	Humidity control	CLIA waiver in progress	[24]
Fe_3_O_4_ Nanozyme Ebola Sensor	EBV Glycoprotein	Plasma	220 fg/mL	<10 min	Plasma separation	Emergency use authorized	[25]
ZnS QD Glucose Sensor	Glucose	Sweat	0.018 mg/dL	<30 s	pH adjustment	ISO 13485-ready	**[26]**
Microfluidic K-Ras Sensor	K-Ras mutation	Buccal swab	1:10,000 ratio	~5 min	DNA extraction	IVD-R compliant	[51]
Screen-Printed Bilharzia Sensor	*S. mansoni* antibodies	Serum	0.1 µg/mL	<15 min	Antigen immobilization	Field trial validated	[58]
IoT-Integrated Lactate Sensor	Lactate	Sweat	0.02 mmol/L	<20 s	Electrolyte balancing	ISO 13485-ready	[30]
SPR Nanoantenna Ebola Sensor	EBV antigen	Plasma	220 fg/mL	<10 min	Refractive index calibration	Emergency use authorized	**[25]**

**Table 6:** Overview of nanosensors, with respect to type, classification, target analytes and performance.

Nanosensor Type/Platform	Classification	Target Analyte	Performance Metrics	Reference
Graphene-based glucose nanosensor	Biological (enzymatic/non-enzymatic)	Glucose	LOD: 0.0054 mg/dL; Linear range: 0.18–900.78 mg/dL	[26,32]
SERS nanosensor	Chemical/Optical	Tumor markers	Signal enhancement > 10^6^; Specificity > 90%; SNR > 30 dB	[27,38,44]
Quantum dot bioconjugates	Biological/Optical	PSA (prostate-specific antigen)	LOD: 1 ng/mL; Imaging accuracy: 93%	[46]
AFM cantilever nanosensor	Physical/Mechanical	BRAF mutation, transcription factors	Sensitivity: 92%; Response time < 5 min	[47,49]
Ebola virus nanosensor (plasmonic nanoantenna)	Biological/Optical	EBV antigen	LOD: 220 fg/mL; Sensitivity: 95.8%; Time-to-result < 10 min	[25]
Wearable cardiac nanosensor	Biological/Electrochemical	BNP, CRP, LDL	Continuous monitoring; Cloud-based transmission	[50]
Microfluidic nucleic acid nanosensor	Biological	K-Ras mutation	Detection at 1:10,000 mutant-to-wild-type ratio	[51]
Nanozyme-strip (Fe_3_O_4_ MNPs)	Biological/Catalytic	Ebola glycoprotein	Colorimetric detection; Rapid point-of-care	[25]
HIV nano-microfluidic sensor	Biological	HIV viral load	Single-particle detection < 10 nm; Rapid viral quantification	[53]
Schistosomiasis AuNP nanosensor	Biological/Immunosensor	Soluble egg antigen (SEA)	Sensitivity: 92%; Specificity: 88%; Cost < $1/test	[58]

## Data Availability

Data are available upon reasonable request from the corresponding author.
